# Women’s impressions of their inpatient birth care as provided by family physicians in the Shizuoka Family Medicine Training Program in Japan

**DOI:** 10.1186/1447-056X-12-1

**Published:** 2013-05-22

**Authors:** Mariko Yokota, Shinji Tsunawaki, Keiichiro Narumoto, Michael D Fetters

**Affiliations:** 1 Shizuoka Family Medicine Training Program, Kikugawa Family Medicine Center, 1055-1 Akatsuchi, Kikugawa, Shizuoka 437-1507, Japan; 2 Department of Family Medicine, Kikugawa Municipal General Hospital, 1632 Higashiyokoji, Kikugawa, Shizuoka 439-0022, Japan; 3 Department of Obstetrics, Gynecology, and Family Medicine, Hamamatsu University, School of Medicine, 1-20-1 Handayama, Higashi-ku, Hamamatsu, Shizuoka 431-3192, Japan; 4 Department of Obstetrics and Gynecology, Kikugawa Municipal General Hospital, 1632 Higashiyokoji, Kikugawa, Shizuoka 439-0022, Japan; 5 Department of Family Medicine, University of Michigan, 1018 Fuller Street, Ann Arbor, Michigan 48104-1213, USA

**Keywords:** Family medicine, Family physicians, Residency training, Obstetrics, Women’s health, Qualitative research, Rural health care, Japan, Health care shortage, Community health

## Abstract

**Background:**

Even though Japan faces serious challenges in women’s health care such as a rapidly aging population, attrition of obstetrical providers, and a harsh legal climate, few family medicine residency training programs in Japan include training in obstetrics, and the literature lacks research on women’s views of intra-partum pregnancy care by family physicians.

**Findings:**

In this exploratory study, we conducted semi-structured qualitative interviews with five women who received their admission, intrapartum, delivery and discharge care from family medicine residents in the obstetrics ward of a community training hospital. Four women had vaginal births, and one had a Cesarean section. Three were primiparous, and two multiparous. Their ages ranged from 22–33. They found value in family physician medical knowledge and easy communication style, though despite explanation, some had trouble understanding the family physician’s scope of work. These women identified negative aspects of the hospital environment, and wanted more anticipatory guidance about what to expect physically after birth, but were enthusiastic about seeing a family doctor after discharge.

**Conclusions:**

These results demonstrate the feasibility of family medicine residents providing inpatient birth care in a community hospital, and that patients are receptive to family physicians providing that care as well after discharge. Women’s primary concerns relate mostly to hospital environment issues, and better understanding the care family physicians provide. This illustrates-areas for family physicians to work for improvements.

## Findings

### Background

Women’s health care faces serious challenges in Japan due to the rapidly aging population and low birth rate [1] and physician trepidation with the legal climate [2]. In addition, changes in the obstetrician workforce, both a decline in student interest in the specialty and a growing proportion of female OB/GYN trainees [3], the decreasing number of facilities providing obstetrical care [4], the trend to centralize obstetrical care into larger centers, and an aging work force [5] contribute to the need for family physicians to train in pregnancy care and provide it after going into practice in Japan.

The family physician’s ability to provide prenatal, pregnancy, post-partum and newborn care offers great potential for helping address these challenges [6]. This is especially true in rural areas and small communities. The low birth rate in low density population areas cannot support a critical mass of full-time obstetrician/gynecologists. These areas face attrition from aging of current obstetricians who retire, drop obstetrics or relocate to larger hospitals for benefits of higher obstetrics volume and lifestyle advantages of group practice. In addition, they face difficulty recruiting recently trained obstetricians as there are fewer of them, and female obstetricians tend to drop obstetrics practice due to exhausting work schedules that compromise their personal lives, or to locate in higher population density areas for personal and professional reasons. The inclusion of pregnancy care by family physicians in such settings has great potential for supporting the practice of obstetrics in rural and small communities.

For family physicians to achieve their full potential in the community [7], training experiences in OB/GYN and pediatrics are prerequisites. While the Japan Primary Care Association [8] had certified 161 programs as of December 18, 2012 to train family physicians, extraordinarily few have actually incorporated training in pregnancy and newborn care.

The Shizuoka Family Medicine (SFM) Residency Program was established in April, 2010 [9] with the unprecedented support of a four-year grant from the Ministry of Health, Labor and Welfare through a community rejuvenation application submitted by the Shizuoka Prefecture [10]. Developed in collaboration with the University of Michigan Department of Family Medicine [11], the SFM program trains family physicians in the full breadth of cradle to grave care. Training occurs in two community hospitals, the Kikugawa Municipal General Hospital and Morimachi Public Hospital each with a separate family medicine clinic, and a tertiary care hospital, Iwata Municipal General Hospital for inpatient pediatrics and obstetrics rotations. In one of the affiliated family medicine clinics, the Kikugawa Family Medicine Center, residents and select faculty provide prenatal care. Resident experiences in providing obstetrical care occur in Kikugawa Municipal General Hospital and Iwata Municipal General Hospital. Residents spend four months during residency training in obstetrics and gynecology, two months each in years one and two. Residents experience a combination of a small number of continuity deliveries and deliveries of women from the hospital-based OB/GYN clinic that occur on the general obstetrics service. Senior family medicine electives in OB/GYN are possible.

#### Aim and objective

The purpose of this study was to elucidate from women who received care at Kikugawa Municipal General Hospital their views about receiving their inpatient birth care from family physician residents. The acceptability of family physicians providing birth care is a very timely and important topic in Japan since there are very few residency programs or family physicians that provide birth care. Moreover, the Shizuoka Family Medicine Residency Training program has just started and there is no precedent of family physician residents participating in birth care in the training hospital itself.

### Methods

#### Design

We conducted an exploratory qualitative study using semi-structured interviews. This approach allows subjects to respond to questions posed by the interviewer and volunteer areas of importance to the subject. The Kikugawa Municipal General Hospital Ethics Committee approved this research.

#### Setting

The Shizuoka Family Medicine Residency Program, Kikugawa City, Japan, an area situated about mid-way between Tokyo and Kyoto, Japan served as the setting for this research [9]. All five women in this project received their prenatal care at the Kikugawa Municipal General Hospital from one of two OB/GYN doctors in the outpatient clinic where family medicine residents also rotate. All inpatient care for participants was provided by two family medicine residents in collaboration with the nurse midwives in the hospital obstetrics ward. The residents bear responsibility to admit patients, round daily, write medication orders and deliver patients with assistance of the nurse midwives—usual practice in Japan—and under the supervision of an attending. All patients including study participants received a handout (available in Japanese upon request) describing family physicians and family medicine resident participation on the hospital obstetrics service.

#### Participants

We sought women participants in the hospital obstetrics ward of the community training hospital who received their admission, intrapartum, delivery and discharge care from a family medicine resident. Consecutive women admitted by a family medicine resident were eligible to participate and approached. All women agreed to participate.

#### Instrument

The interview instrument was designed for the interviewer to first explain that the purpose of the research was to gain a broad understanding of the participants’ perspectives on their birth care from family medicine residents, and gain insight into ways for family physicians to improve woman’s birth experiences based on their views. The interviewer then asked participants four interview questions, namely, “What went well?”, “What went poorly?”, “What are areas of improvement?”, and “Other general impressions?”

Data collection procedures: Individuals gave verbal consent to participate. The interviews were conducted during morning rounds by a family medicine resident directly with the mothers in their hospital room one to five days after delivery. The hospitalization duration for normal delivery routinely lasts six days in this hospital. Participant comments were recorded by the interviewer.

#### Analysis

For the analysis, we employed a template approach [12], i.e., the text was analyzed and edited by the four questions that comprised the template. We organized the results to develop a narrative of the participant’s views.

### Results

Participating women’s ages ranged from 22 to 33, three were primiparous, and two were multiparous. One delivered by Cesarean section while the rest delivered vaginally. As illustrated in Table 1, our results follow the structure of the interview questions, namely, positive and negative experiences, areas for improvement, and other general impressions.

**Table 1:** Responses about inpatient birth care by family physician residents

**Positive experiences**	**Negative experiences**	**Areas for improvement**	**General impressions**
• Family physician’s role between that of a midwife and obstetrician	• Lack of information provided about what to expect after the birth	• Need for information about post-partum physical discomfort, i.e., pain from the surgical incision or intravenous catheter, postpartum abdominal pain, and/or breast engorgement	• Wanting follow-up care by a family doctor
			• Wanting the family doctors involved in their birth care to become their regular doctors
• Feeling safe			
	• Lack of relaxing atmosphere in the delivery room		
• Friendliness of family physicians encouraged women to talk with them			
	• Uncertainty about what a family physician does can cause anxiety		
			• Being glad that a family doctor was there
		• Need to improve the delivery room environment with background music, adjustable lights and pleasant aroma	
		• Need to provide an explanation about what family physicians do during prenatal care or at the time of admission	

### Positive experiences

One positive experience highlighted the family medicine resident’s functioning between that of a nurse midwife and an obstetrician. While nurse midwives are usually available, they do not have the same degree of medical knowledge as a family physician about physiology and anatomy. Thus, the family medicine residents could provide more complete answers to the women’s questions. While obstetricians are knowledgeable especially about the physiological and surgically-relevant aspects of birth, the women in this study could meet only briefly with the obstetrician about once per day. In contrast, the family medicine residents participating in this research were present throughout the day and could answer the women’s questions. Regarding their care, patients had various comments. For example, they described care as feeling “safe” since family physicians provided repeated examinations and were present during the labor period, or they shared that it was easy to speak with a family physician.

### Negative experiences

Women offered few comments about negative aspects of their care. Specifically, they voiced the need for more information about what to expect after the birth relative to pain and bleeding. One woman shared that the environment of the delivery room made her nervous. They also expressed feeling some anxiety about not understanding a family physician’s role and scope of care.

### Areas for improvement

Areas for improvement focused on how to ameliorate the problems raised. Thus, participants wanted more information about how long there would be incisional pain, postpartum abdominal pain, breast engorgement, and pain from the site of the intravenous catheter. Measures to improve the environment could include playing music, using lights with adjustable brightness, and eliminating odors in the ward. Also, to calm patients, a verbal and written explanation during prenatal care or at the time of admission that describes the role and expertise offered by family physicians would be helpful.

### General impressions

Overall impressions about family physicians included: wanting to be seen at follow-up by a family physician, wishing for a family physician to become the regular doctor, and feeling glad that a family physician was there during birth care.

### Conclusions

Though this was an exploratory study, these findings demonstrate the feasibility of family physicians working together with obstetricians and nurse midwives in rural family medicine residency hospital settings. As illustrated, patients feel there are benefits due to knowledge differences between family medicine physicians versus other types of physicians and due to the broad scope of family medicine training. While the concept of a family physician was new to them, after the family physician’s role and training were clarified, the participants could appreciate the value of family physicians’ contributions to their birth care and anticipate benefits of continuity between the hospital and the outpatient setting. These data illustrate that Japanese women find hospital-based birth care by family physicians as acceptable, and should provide encouragement for family medicine residents to train in birth care in Japan.

While this research was designed to assess women’s views about family physician involvement in their hospital-based care, most negative comments referred to concerns and possible improvements in the hospital environment. To address these findings, the family physician’s view of patient-centered maternity care – when applied in this setting – could help improve the quality of care. Anticipatory guidance, that is, anticipating the patient’s experience and explaining what will happen beforehand, is a critical communication skill strongly emphasized in family medicine training that could address concerns about post-discharge care.

Even though family medicine residents used a handout to explain in person what a family physicians does at the time of admission, and what they would be doing during the hospitalization, women still found it difficult to understand the family physician’s scope of work. As few patients have yet experienced the care of a family physician, it will likely take time for patients to come to understand the breadth and depth of family medicine. Pamphlets, use of social media such as Facebook, and community campaigns about family medicine might help. We are encouraged by the subjects who indicated their interest in seeing a family doctor after the hospitalization. If experience from the US applies, patients who experience the comprehensive scope of medical skills and care offered by a family physician will communicate this knowledge to their friends, and as a result, the popularity of family physicians will grow by word-of-mouth.

As an exploratory study, this research had only a small number of participants, though the encouraging nature of these data are compelling. Further exploration can address many additional issues regarding roles of family physicians in the entire pregnancy experience including prenatal and post-partum care. While it is plausible participants had other concerns but were reluctant to express them face-to-face with their doctors, we believe most participants answered candidly as suggested by both their positive and negative comments. Future research also could include women’s perspectives regarding specific ways to improve the training program for family medicine residents as this was beyond the scope of this research.

While most family medicine training programs in Japan have not embraced training in birth care, we believe that even if a family medicine resident does not provide birth care after completion of residency, the birth experience during training will enable family physicians to understand the nature of the birth process from the patient’s perspective, to grasp many issues that occur at the beginning of life and to provide anticipatory guidance to their patients about birth issues [6]. These preliminary findings demonstrate the feasibility of family physician residents providing prenatal, intra-partum and post-partum care, difficulties patients have distinguishing between hospital and family physician influences on their care, and positive contributions family physician residents can make in women’s hospital birth experiences.

## Abbreviations

JAFM: The Japanese academy of family medicine; MHLW: Ministry of health, labour and welfare; OB/GYN: Obstetrics and gynecology; SFM: Shizuoka family medicine; SMARTER FM: Shizuoka-University of Michigan advanced residency training, exchange and research in family medicine project.

## Competing interests

The authors declare they have no competing interests.

## Authors’ contributions

MY conceptualized the design of the study, carried out the primary data collection and worked on the preliminary write-up. ST contributed to the design and data collection support. KN helped conceived the study and led the analysis. MF assisted in the data analysis and primary mentoring for writing the paper. All authors contributed to reading, editing, and approval of the final manuscript.

## Author’s information

Mariko Yokoto currently is a family medicine resident post-graduate year five (including a two-year rotating internship), who began this study during her first year of family medicine training. Shinji Tsunawaki currently is a PGY6, who supervised Dr. Yokota on the hospital ward and now serves as a fellow in the SFM family medicine training program. Keiichiro Narumoto serves as faculty member, SFM program and Kikugawa Municipal General Hospital, Department of Obstetrics and Gynecology, and as a local mentor to Drs. Yokota and Tsunawaki. Michael D. Fetters serves as Director of the Japanese Family Health Program, and is Professor at the University of Michigan, Department of Family Medicine. Dr. Fetters also serves as the Principal Investigator (PI) on the Shizuoka-University of Michigan Advanced Residency, Training, Education and Research in Family Medicine (SMARTER FM) project [9] that supports the University of Michigan’s collaboration in the launch of the SFM program.
